# Autoantibody against transient receptor potential M1 cation channels of retinal ON bipolar cells in paraneoplastic vitelliform retinopathy

**DOI:** 10.1186/1471-2415-12-56

**Published:** 2012-11-13

**Authors:** Yujuan Wang, Mones S Abu-Asab, Wei Li, Mary E Aronow, Arun D Singh, Chi-Chao Chan

**Affiliations:** 1 Section of Immunopathology, Laboratory of Immunology, National Eye Institute, National Institutes of Health, 10 Center Dr., 10/10N103, NIH/NEI, Bethesda, MD, 20892-1857, USA; 2 Zhongshan Ophthalmic Center, Sun Yat-sen University, Guangzhou, China; 3 Unit on Retinal Neurophysiology, National Eye Institute, National Institutes of Health, Bethesda, MD, USA; 4 Department of Ophthalmic Oncology, Cole Eye Institute, Cleveland Clinic, Cleveland, OH, USA

**Keywords:** Paraneoplastic vitelliform retinopathy, Autoimmune retinopathy, Transient receptor potential channel, Bipolar cell, Melanoma-associated retinopathy, Autoantibody

## Abstract

**Background:**

Paraneoplastic retinopathy is caused by the cross-reaction of neoplasm-directed autoantibodies against retinal antigens and results in retinal damage. Paraneoplastic vitelliform retinopathy, a presumed paraneoplastic retinopathy with features of atypical melanoma-associated retinopathy, has recently been reported in patients with metastatic melanoma. Ocular ultrastructure and its autoantibody localization of paraneoplastic vitelliform retinopathy are still indefinable. This is the first report of anti-transient receptor potential M1 antibody directly against human retinal bipolar dendritic tips in a melanoma patient with paraneoplastic vitelliform retinopathy.

**Case presentation:**

We present a pair of postmortem eyes of an 80-year-old male with metastatic cutaneous melanoma, who developed paraneoplastic vitelliform retinopathy. The autopsied eyes were examined with light microscopy, immunohistochemistry, and transmission electron microscopy. Microscopically, the inner nuclear layer and outer plexiform layer were the most affected retinal structures, with local thinning. The lesions extended to the outer nuclear layer, resulting in focal retinal degeneration, edema, and atrophy. No active inflammation or melanoma cells were observed. Immunohistochemistry showed tightly compact bipolar cell nuclei (protein kinase C alpha/calbindin positive) with blur/loss of ON bipolar cell dendritic tips (transient receptor potential M1 positive) in diffusely condensed outer plexiform layer. The metastatic melanoma cells in his lung also showed immunoreactivity against transient receptor potential M1 antibody. Transmission electron microscopy illustrated degenerated inner nuclear layer with disintegration of cells and loss of cytoplasmic organelles. These cells contained many lysosomal and autophagous bodies and damaged mitochondria. Their nuclei appeared pyknotic and fragmentary. The synapses in the outer plexiform layer were extensively degenerated and replaced with empty vacuoles and disintegrated organelles.

**Conclusion:**

This case provides a convincing histological evidence of melanoma-associated autoantibodies directly against transient receptor potential M1 channels that target the ON bipolar cell structures in the inner nuclear and outer plexiform layers in paraneoplastic vitelliform retinopathy.

## Background

Paraneoplastic retinopathy (PR) is mediated by tumor-associated autoantibodies directed against retinal antigens. PR patients usually develop reduced visual acuity, photopsias, nyctalopia, visual field defects, and reduced a- and/or b-waves in electroretinography (ERG) [1,2]. The two main types of PR are cancer-associated retinopathy (CAR) and melanoma-associated retinopathy (MAR). CAR is mostly associated with small-cell lung cancer and has specific autoantibodies against photoreceptors [3,4]. MAR typically develops in patients with metastatic cutaneous or uveal melanoma; autoantibodies against retinal bipolar cells are the hallmark of MAR [5-8]. In MAR, the fundus is unremarkable in 44% cases, although fundus changes such as focal depigmentation, optic nerve pallor, and/or retinal vascular attenuation have been reported [6,9]. Recently, a MAR-like retinopathy phenotype, termed paraneoplastic vitelliform retinopathy, has been recognized that is also associated with cutaneous or uveal melanoma [10,11]. Unlike the typical normal-appearing fundus in MAR, paraneoplastic vitelliform retinopathy is characterized by vitelliform retinal lesions at the level of the retinal pigment epithelium (RPE) with associated serous retinal detachment and subretinal accumulation of hyperautofluorescent material in the posterior pole [10,12].

Similar to CAR and MAR that are immunologically heterogeneous, serum autoantibodies, such as anti-bipolar cell antigens, interphotoreceptor retinoid-binding protein (IRBP), carbonic anhydrase II (CAII), and bestrophin, are reported in patients with paraneoplastic vitelliform retinopathy [10,13,14]. Among them, anti-bipolar cell antibody is the most commonly identified autoantibody [15,16]. As reduced ERG b-wave is indicative of retinal bipolar cell impairment, it is hypothesized that the autoantibodies generated by melanoma patients are directed against retinal bipolar cells in paraneoplastic vitelliform retinopathy.

Recently, a new connection between PR and bipolar cells has been made by the identification of a cation channel named transient receptor potential M1 (TRPM1, also known as melastatin 1). TRPM1 was initially identified in melanocytes and cutaneous melanoma and is a marker for metastasis and prognosis [17-19]. The loss or downregulation of *TRPM1* mRNA correlates with an increased risk of metastatic melanoma [17,18]. The patterns of *TRPM1* transcript expression also help differentiate Spitz nevi from nodular melanomas, with higher ubiquitous expression in Spitz nevi and higher incidence of loss in nodular melanomas [17]. TRPM1 is also expressed in retinal bipolar dendritic tips [20-23]. Several studies demonstrated that TRPM1 cation channel is essential for ON bipolar cell signaling [22,24,25]. In addition, several groups have found that human *TRPM1* mutations are linked to congenital stationary night blindness [26-28]. Furthermore, it has been reported that TRPM1 is the target of autoantibodies in some PR patients [29,30].

As a subtype of PR, the exact pathogenesis of paraneoplastic vitelliform retinopathy remains elusive. Recently, we reported the clinical manifestations and pathology of a paraneoplastic vitelliform retinopathy case with lesions in inner nuclear layer (INL), outer plexiform layer (OPL), and outer nuclear layer (ONL) of the retina, the loci of which correspond to the clinical fundus lesions [16]. Herein, we re-examine this case including metastatic melanoma cells in the lung with immunohistochemistry and transmission electron microscopy (TEM).

## Case presentation

### Medical history

The detailed clinical history and some pathological findings were described previously [16]. Briefly, an 80-year-old male with lung biopsy confirmed metastatic melanoma was suspected of choroidal metastases. One year after diagnosis with metastatic melanoma, he developed nyctalopia and bilateral retinal lesions. The patient was referred to the Department of Ophthalmic Oncology at the Cole Eye Institute. His best corrected visual acuity was 20/30 in the right eye and 20/25 in the left eye. Fundus examination showed bilateral, multiple, deep, yellowish lesions in the posterior pole and mid-periphery. Optical coherence tomography excluded the possibility of choroidal metastases. ERG showed a mild reduction in both a- and b-wave amplitudes for both scotopic and photopic waveforms. His serum was found to have autoantibodies against CAII and an unknown 35-kDa RPE protein. The patient expired approximately one month following ophthalmic examination.

### Pathological findings

**Figure 1:**
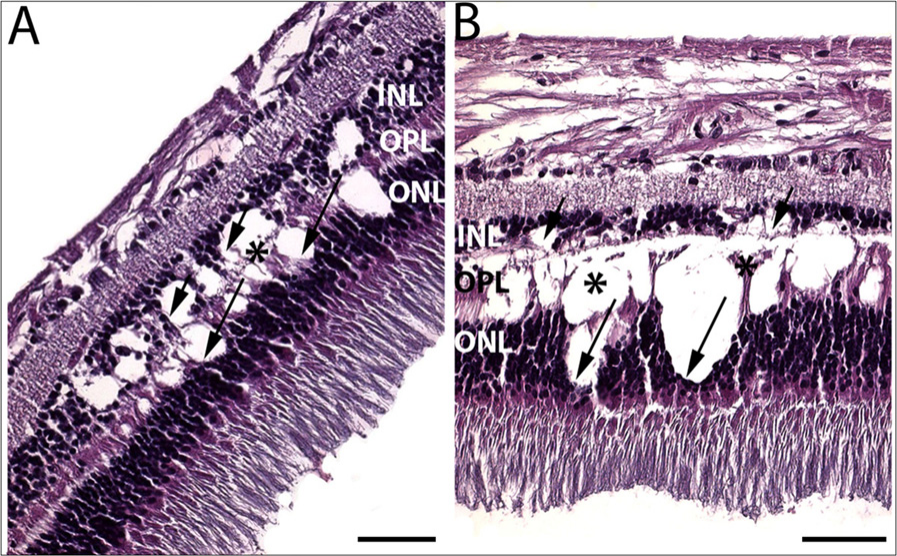
**Photomicrograph of retinal lesions in a case of paraneoplastic vitelliform retinopathy.** (**A**) Early-stage retinal lesions with focal edema and splitting in the inner nuclear layer (INL, short arrows) and outer nuclear layer (ONL, long arrows); mild atrophy of outer plexiform layer (OPL) is also visible (asterisk). (**B**) Late-stage retinal lesions with severe atrophy/loss of OPL (asterisks); the lesion extends from INL (short arrows) and OPL (asterisks) toward ONL (long arrows) regions (Hematoxylin and eosin, original magnification, ×200, scale bar, 50 μm).

Gross examination showed two round, yellowish-white deep retinal lesions barely visualized along the inferior arcade temporal to the macula of the left eye [16]. Microscopically, focal edema, separation, and atrophy were observed in the INL, extending to the OPL and ONL (Figure 1). Neither active inflammatory infiltrates nor melanoma cells were observed.

### Immunohistochemical findings

**Figure 2:**
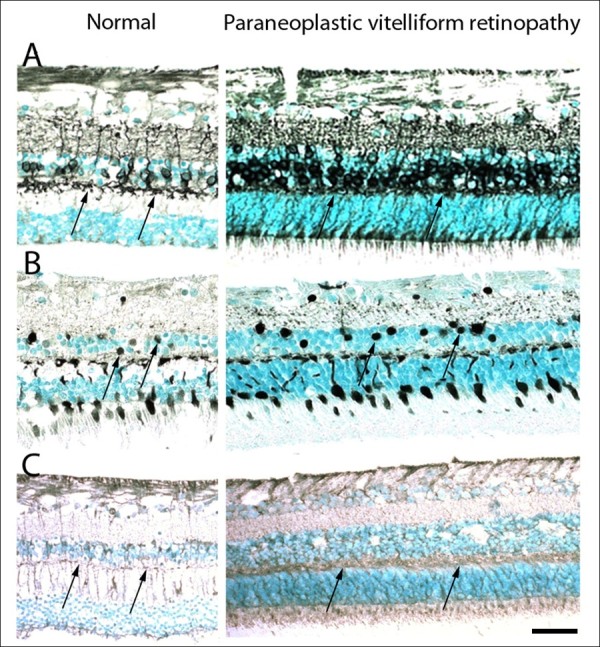
**Photomicrograph of immunostaining in normal retina and relatively intact areas of the paraneoplastic vitelliform retinopathy case.** (**A**) Protein kinase C alpha labeled bipolar cells are tightly packed with blurred dendritic structures (arrows) and condensed outer plexiform layer (OPL). (**B**) Calbindin positive bipolar cells (arrows) are slightly packed with thinned and reduced OPL. (**C**) Transient receptor potential M1 labeled ON bipolar cell dendritic tips (arrows) had blurred structures and loss of puncta that appears in normal human retina (Avidin-biotin-complex immunohistochemistry, original magnification, ×200, scale bar, 50 μm).

**Figure 3:**
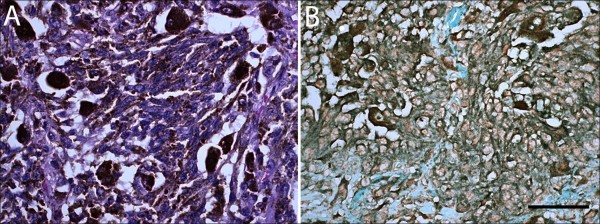
**Photomicrograph of immunostaining in metastatic lung melanoma of a case with paraneoplastic vitelliform retinopathy.** (**A**) Metastatic melanoma cells infiltrate in the lung tissue (Hematoxylin and eosin, original magnification, ×400). (**B**) Transient receptor potential M1 labeled melanocytes are observed in the lung metastasis (Avidin-biotin-complex immunohistochemistry, original magnification, ×400, scale bar, 100 μm).

The antibodies for immunohistochemistry are listed in Table 1. An age-matched normal human retina and relatively normal areas of the paraneoplastic vitelliform retinopathy case were compared. In normal eye, different types of retinal bipolar cells in the INL are stained positively with protein kinase C alpha (PKCα, Figure 2A) or calbindin, a calcium binding protein (Figure 2B); however, the nuclei of these labeled bipolar cells were tightly compact in this case. The thickness of the OPL was greatly reduced (nearly 1/3 thickness of OPL in the normal eye) and the PKCα - positive dendritic structures appeared blurred (Figure 2A). TRPM1 staining demonstrated a specific loss of puncta in the OPL of this case; in contrast, the TRPM1-labeled dendritic tips of ON bipolar cells in normal eye are distinct and well defined, located near the INL in the OPL (Figure 2C). The immunoreactivities were similar in both eyes. There were many metastatic melanoma cells infiltrated the lung (Figure 3A), and these malignant cells were also stained positively with TPRM1 antibody (Figure 3B).

**Table 1:** Cellular markers used in the study

**Antibody**	**Size**	**Immunoreactivity**
PKCα	82 kDa	Rod bipolar cell, DB4 ON bipolar cell
Calbindin	28 kDa	DB3 OFF bipolar cell
TRPM1	182 kDa	ON bipolar dendrite, melanocyte

PKC= protein kinase C; DB=diffuse bipolar cells; TRPM1=transient receptor potential M1.

### TEM findings

**Figure 4:**
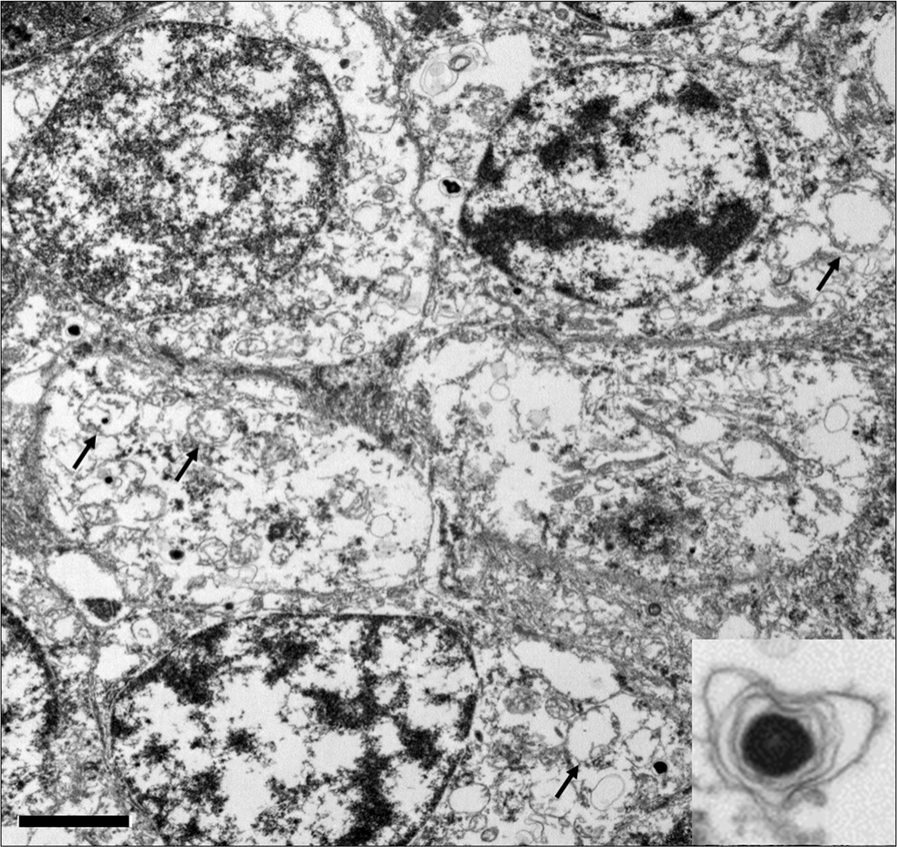
**Transmission electron microscopy photomicrograph of inner nuclear layer of retina in paraneoplastic vitelliform retinopathy.** The degenerated cells are at various stages of disintegration and most of their cytoplasmic organelles are absent. The mitochondria were degenerate and only their outer membranes were visible (arrows, original magnification, ×8000, scale bar, 2 μm). There were numerous lysosomal and autophagous bodies (Figure 4 inset, original magnification, ×25000) within the cells.

TEM showed abnormal cellular structures in the INL and OPL of the retina. The INL was characterized with cytoplasmic degeneration and cellular disintegration at different stages (Figure 4). The nuclei contained chromosomal fragmentation. Many cells in the INL contained damaged mitochondria, numerous lysosomal and autophagous bodies in their cytoplasm (Figure 4 & inset). Abundant empty vacuoles, disintegrated mitochondria, fragmented rough endoplasmic reticulum, prominent glial filaments, and many apoptotic bodies were admixed with the deteriorated synapses in the OPL.

## Discussion

Our study provides direct morphological evidence that the retinal bipolar cells are damaged in paraneoplastic vitelliform retinopathy. Importantly, TRPM1 is identified as a target of anti-bipolar antibody produced in this patient with paraneoplastic vitelliform retinopathy. TRPM1 channels on the ON bipolar dendritic tips in the OPL were specifically targeted and TRPM1 antigens were also detected in the metastatic melanoma cells in the lung. Additionally, the ultrastructures of the bipolar nuclei and synapses were damaged.

In addition to previously reported serum autoantibodies, including IRBP, CAII, and bestrophin in patients with paraneoplastic vitelliform retinopathy [10,13,14], peroxiredoxin 3 (PRDX3) is identified as a new putative antigen in RPE attacked by autoantibodies in a melanoma patient with paraneoplastic vitelliform maculopathy [12]. It is hypothesized that the paraneoplastic autoimmune reaction against this 26-kDa PRDX3 may be a cause of paraneoplastic vitelliform retinopathy. However, our patient’s serum was only detected two retinal autoantibodies against a 30-kDa protein (CAII) and an unknown 35-kDa RPE protein by Western blot [16].

Visual processing in human retina is accomplished by several types of neurons. Bipolar cells in the INL are the second-order neurons that play an essential role in visual signal transduction. Other cells in the INL that are related to bipolar cell signaling processing include amacrine, horizontal, and Müller cells. Their detailed function and cell markers are summarized in Table 2. Through complicated synaptic interactions, bipolar cell dendrites receive information from the photoreceptors and horizontal cells and transmit these signals to the ganglion and amacrine cells. Based on the two types of photoreceptors (rods and cones) that they connect to, bipolar cells are divided into one rod subtype (ON type) and 8 cone subtypes (ON and OFF types). Light hyperpolarizes photoreceptors and reduces glutamate release from photoreceptors. The reduced glutamate levels depolarize ON bipolar cells and hyperpolarizes OFF bipolar cells. The opposite responses of bipolar cells are mediated by different glutamate receptors (GluRs) expressed on ON (metabotropic GluR, mGluR6) and OFF (ionotropic GluRs) bipolar dendrites [31,32]. Responses of photoreceptors and bipolar cells are reflected in ERG a- and b- waves, respectively. Unlike ionotropic GluRs on OFF bipolar cells, which are ion-permeable channels themselves, mGluR6 receptors on ON bipolar cells are G-protein coupled receptors that are separated from the channels that they gate [33]. Recently, TRPM1 has been identified to be the cation channel that is downstream to the mGluR6 cascade of ON bipolar cells [22,24].

**Table 2:** Neuron cells in the inner nuclear layer and their functions and markers

**Cell type**	**Function**	**Cell marker**
Bipolar	Responsible for signals transmitting from photoreceptors to retinal ganglion cells; divided into ON and OFF bipolar cells according to their responses to visual stimuli: light stimuli increase the conductance of ON bipolar cells but decrease that in OFF bipolar cell	**ON bipolar:** PKCα (rod, DB4), CD15 (DB6), Islet-1, Glycine, Goα [34]; **OFF bipolar:** Recoverin, Glutamate transporter-1(DB2), PKCβ, Calbindin (DB3) [34]; **ON and OFF bipolar:** CaB5 [34]
Amacrine	Responsible for modulation of signal reaching ganglion cells; affecting orientation selectivity, light–dark effect and color discrimination	Syntaxin [35], Dab1, AP2α [36], Pax6 [37],
Horizontal	Having an integrative role in retinal processing and release inhibitory neurotransmitters; providing feedback for the photoreceptors	Parvalbumin [38]
Müller	Forming architectural support structures in the retina; involved in protein synthesis, intracellular transport and secretion; helping nourish and maintain the outer neuroretina	Glutamine synthetase [39]

PKC=protein kinase C; DB= diffuse bipolar cells; CaB5=calcium-binding protein 5; Dab1=Disabled-1; AP2α=activator Protein 2 alpha; Pax6=paired box.

Recently, TRPM1 is identified as a suspected target of the autoantibodies produced in melanoma patients with MAR [30]. Dhingra et al. [29] demonstrate that sera of melanoma patients with PR specifically recognized TRPM1 in mouse bipolar cells. Up to date, no TRPM1 abnormality has been illustrated in the human retina of paraneoplastic vitelliform retinopathy. Although the clinical findings of PR patients with serum anti-TPRM1 autoantibody have reported in the literature (CAR and MAR patients) [29,30], the pathological mechanism of all these PR entities is through the common identified TRPM1 channel. Additional unknown mechanisms may play a role in determining the phenotypes of PR patients. In this study, we illustrate that TRPM1 channels are diffusely damaged and TRPM1 antigen is also expressed on the metastatic lung melanoma cells. Although downregulation or loss of *TRPM1* expression has been reported in melanoma metastasis [18], this study cannot compare TRRM1 expression levels between original cutaneous melanoma and lung metastasis in this case due to lack of original melanoma specimens. However, our findings strongly support that anti-TRPM1 autoantibody is involved in the pathogenesis of paraneoplastic vitelliform retinopathy, an atypical MAR. ERG of *Trpm1* deficient mice has a normal a-wave, but no b-wave, indicating a loss of bipolar cell response [24]. The damaged TRPM1 channel, either before or after ON bipolar cell damage, could cause abnormal light signal transduction, which consequently affects night vision and ERG b-wave. In this case, lesions were also found from the INL and OPL extending to the ONL that might cause abnormal ERG a-wave. However, whether the ONL changes are primary or secondary events remains to be resolved.

## Conclusions

Both immunohistochemistry and TEM showed specific damages in bipolar cells and TRPM1 cation channel on the ON bipolar dendritic tips. The immunohistochemical data provide evidence of autoantibodies against the TRPM1 cation channel on retinal ON bipolar cells and metastatic melanoma cells in paraneoplastic vitelliform retinopathy.

## Consent

Written informed consent was obtained from the patient’s family for publication of this Case report and any accompanying images. A copy of the written consent is available for review by the Series Editor of this journal.

## Abbreviations

PR: Paraneoplastic retinopathy; ERG: Electroretinography; CAR: Cancer-associated retinopathy; MAR: Melanoma-associated retinopathy; RPE: Retinal pigment epithelium; IRBP: Interphotoreceptor retinoid-binding protein; CAII: Carbonic anhydrase II; TRPM1: Transient receptor potential M1; INL: Inner nuclear layer; OPL: Outer plexiform layer; ONL: Outer nuclear layer; TEM: Transmission electron microscopy; PKCα: Protein kinase C alpha; PRDX3: Peroxiredoxin 3; GluRs: Glutamate receptors.

## Competing interests

The authors declare that they have no competing interests.

## Authors’ contributions

YW drafted the manuscript, participated in acquisition of data (immunohistochemistry), collection of data, analysis and interpretation of data, and final approval of the version to be published. MSA participated in acquisition of data (electron microscopy), collection of data, data analysis, data interpretation, and revising the manuscript. WL participated in substantial contribution to conception and design, data analysis, data interpretation, and revising the manuscript critically. MEA participated in acquisition of data (clinical ophthalmology), data collection, data analysis and interpretation of data. ADS participated in acquisition of data (clinical ophthalmology), collection of data, data analysis, and interpretation of data. CCC substantially contributed to conception and design, acquisition, analysis and interpretation of data, participated in revising the manuscript critically, final approval of the version to be published, and general supervision of the research project. All authors read and approved the final manuscript.

## Pre-publication history

The pre-publication history for this paper can be accessed here:

http://www.biomedcentral.com/1471-2415/12/56/prepub
